# RhoA/ROCK Signaling and Pleiotropic α_1A_-Adrenergic Receptor Regulation of Cardiac Contractility

**DOI:** 10.1371/journal.pone.0099024

**Published:** 2014-06-11

**Authors:** Ze-Yan Yu, Ju-Chiat Tan, Aisling C. McMahon, Siiri E. Iismaa, Xiao-Hui Xiao, Scott H. Kesteven, Melissa E. Reichelt, Marion C. Mohl, Nicola J. Smith, Diane Fatkin, David Allen, Stewart I. Head, Robert M. Graham, Michael P. Feneley

**Affiliations:** 1 Victor Chang Cardiac Research Institute, Darlinghurst, Australia; 2 Cardiology Department, St Vincent’s Hospital, Darlinghurst, Australia; 3 Faculty of Medicine, University of New South Wales, Sydney, Australia; 4 Physiology Department, University of Sydney, Sydney, Australia; 5 Physiology Department, University of New South Wales, Sydney, Australia; Loyola University Chicago, United States of America

## Abstract

**Aims:**

To determine the mechanisms by which the α_1A_-adrenergic receptor (AR) regulates cardiac contractility.

**Background:**

We reported previously that transgenic mice with cardiac-restricted α_1A_-AR overexpression (α_1A_-TG) exhibit enhanced contractility but not hypertrophy, despite evidence implicating this Gα_q/11_-coupled receptor in hypertrophy.

**Methods:**

Contractility, calcium (Ca^2+^) kinetics and sensitivity, and contractile proteins were examined in cardiomyocytes, isolated hearts and skinned fibers from α_1A_-TG mice (170-fold overexpression) and their non-TG littermates (NTL) before and after α_1A_-AR agonist stimulation and blockade, angiotensin II (AngII), and Rho kinase (ROCK) inhibition.

**Results:**

Hypercontractility without hypertrophy with α_1A_-AR overexpression is shown to result from increased intracellular Ca^2+^ release in response to agonist, augmenting the systolic amplitude of the intracellular Ca^2+^ concentration [Ca^2+^]_i_ transient without changing resting [Ca^2+^]_i_. In the *absence* of agonist, however, α_1A_-AR overexpression *reduced* contractility despite unchanged [Ca^2+^]_i_. This hypocontractility is not due to heterologous desensitization: the contractile response to AngII, acting via its Gα_q/11_-coupled receptor, was unaltered. Rather, the hypocontractility is a pleiotropic signaling effect of the α_1A_-AR in the absence of agonist, inhibiting RhoA/ROCK activity, resulting in hypophosphorylation of both myosin phosphatase targeting subunit 1 (MYPT1) and cardiac myosin light chain 2 (cMLC2), reducing the Ca^2+^ sensitivity of the contractile machinery: all these effects were rapidly reversed by selective α_1A_-AR blockade. Critically, ROCK inhibition in normal hearts of NTLs without α_1A_-AR overexpression caused hypophosphorylation of both MYPT1 and cMLC2, and rapidly reduced basal contractility.

**Conclusions:**

We report for the first time pleiotropic α_1A_-AR signaling and the physiological role of RhoA/ROCK signaling in maintaining contractility in the normal heart.

## Introduction

Heart failure is a major cause of death, disability and escalating health costs worldwide as populations age. Inotropic drugs are useful short term, but their long term use may increase mortality, in part due to increased calcium (Ca^2+^) release within cardiomyocytes (CMs). Sympathetic regulation of contractility reflects catecholamine stimulation of CM adrenergic receptors (ARs). β_1_-AR effects are normally dominant. In heart failure, however, β-ARs are downregulated and uncoupled from G proteins, and α_1_-ARs may act to maintain contractility.

We reported previously that transgenic mice with cardiac-restricted α_1A_-AR overexpression (α_1A_-TG) display hypercontractility that is proportional to receptor number, is inhibited by selective α_1A_-AR blockade, and is not due to β-AR cross-talk [1]. Surprisingly, this hypercontractility is not associated with cardiac hypertrophy [1], [2]. Mice with 66-fold overexpression exhibit improved survival after pressure overload [3] or myocardial infarction [4], but 112-fold and 170-fold overexpression reduced survival due to sudden cardiac death consistent with Ca^2+^ overload [5].

Here, we demonstrate that the mechanism underlying the enhanced cardiac contractility of α_1A_-TG mice is indeed an agonist-induced increase in intracellular Ca^2+^ release. In the absence of agonist, however, contractility is *reduced.* This unexpected finding was not due to reduced intracellular Ca^2+^ concentration [Ca^2+^]_i_ but to reduced Ca^2+^ sensitivity of the myofilaments resulting from inhibition of the RhoA/Rho kinase (ROCK) signaling pathway. This inhibition results in hypophosphorylation of myosin phosphatase target subunit 1 (MYPT1), relieving its inhibition of myosin light chain phosphatase (MLCP) and leading to inactivation of cardiac myosin light chain 2 (cMLC2), a key myofilament protein mediating contraction. The inhibition of RhoA/ROCK signaling was mediated by the α_1A_-AR in the absence of ligand, consistent with spontaneous receptor isomerization to a conformation distinct from that which activates increased Ca^2+^ release in the presence of ligand, indicating pleiotropic receptor signaling.

Critically, the regulation of basal contractility by the RhoA/ROCK pathway is shown to be physiologically relevant because its inhibition in non-transgenic mice with normal receptor expression caused a significant reduction in basal contractile function. Because modulation of cMLC activity can increase contractility without altering [Ca^2+^]_i_, the RhoA/ROCK signaling pathway may be a suitable target for development of novel inotropic interventions.

## Materials and Methods

### Animals

The α_1A_-TG mice with cardiac-restricted α_1A_-AR overexpression, established and bred with FVB/N, have been described in detail [1]. Notably, this model is based on overexpression of the wild type α_1A_-AR, not a mutant, thus avoiding concerns of promiscuous activation of unrelated pathways. Male heterozygous α_1A_-TG mice (170-fold overexpression) and their non-transgenic littermates (NTL) aged 8 to 10 weeks were used for this study. Experimental procedures were approved by the Garvan Institute of Medical Research/St Vincent’s Hospital Animal Ethics Committee in accordance with the guidelines of the Australian Code of Practice for the Care and Use of Animals for Scientific Purposes.

### Excitation-contraction Coupling

CMs were isolated by enzymatic retrograde infusion [2]. CMs were examined in a cell bath superfused with gassed Krebs-Henseleit solution with 1.25 mM Ca^2+^ at 32°C. CMs loaded with the fluorescent [Ca^2+^]_i_ indicator Indo-1/AM (1.2 µM) were field-stimulated at 0.5 Hz, and [Ca^2+^]_i_ obtained from photomultipliers. Simultaneously, shortening was measured by edge detection (240 Hz, MyoCam, IonOptix, MA). The concentration-response to phenylephrine (PE, Sigma, Australia) was recorded.

### Isolated Perfused Contracting Heart Preparation

Hearts were excised into ice-cold modified Krebs-Henseleit perfusion buffer. The aorta was perfused at 80 mmHg with perfusion buffer equilibrated with 95% O_2_ and 5% CO_2_ at 37°C and pH 7.4. A fluid-filled balloon was inserted via the mitral valve, and inflated to a diastolic pressure of ∼5 mmHg. Hearts were maintained at 37±0.1°C in a water-jacketed bath. Experiments, performed in separate groups, were: 1) perfusion with successively increasing concentrations of A61603 (0.1 nM−1.0 µM; Sigma, Australia), a selective α_1A_-AR agonist; 2) before and after perfusion with angiotensin II (AngII, 100 nM, 10 min, MP Biomedicals, Australia) or with one of two selective α_1A_-AR antagonists, RS100329 (50 nM, 10 min, Sigma, Australia) or KMD3213 dihydrobromide (100 nM, 10 min, Kissei Pharmaceutical Co. Matsumoto, Japan); 3) for contractile protein measurements, perfusion with saline or RS100329 (50 nM, 8 min), then snap-frozen (liquid nitrogen); 4) for RhoA/ROCK pathway, perfusion with saline or Y-27632, a selective ROCK inhibitor (1 µM, 5 min, Merck Millipore, MA), then snap-frozen; 5) for RhoA/ROCK signaling in agonist-induced responses, A61603 (0.1 nM−1.0 µM) in absence or presence of Y-27632.

### Calcium Sensitivity of Skinned Cardiac Fibers

Skinned left ventricular fiber strips were prepared as described previously [3]. Strips were skinned by immersion in 3% Triton X-100 for 30 min. Strips were activated with a series of solutions of increasing [Ca^2+^] [3].

### Myofilament and Related Proteins and Phosphorylation Status

Steady state levels of the following were determined by Western blot analysis: cardiac troponin I (cTnI) and its Ser23/24 and Ser43 phosphorylated forms (p-cTnI); cTnC; cTnT; cMLC2 and its Ser20 phosphorylated form (p-cMLC2); MYPT1 and its Thr696 phosphorylated form (p-MYPT1); myosin light chain kinase (MLCK); protein kinase Cα (PKCα) and PKCε. Left ventricular tissue was lysed in a buffer (50 mM Tris HCl, 150 mM NaCl, 1% Triton X-100, 1 mM sodium orthovanadate, and 1 mM β-glycerophosphate, 1 mM DTT and protease inhibitor [P8340, Sigma]), homogenized, and proteins quantified using the Pierce BCA Protein Assay Kit. Protein (40 µg) was separated by SDS-PAGE, and transferred to PVDF membranes (Bio-Rad Laboratories) blocked for 2 hours at room temperature with 5% bovine serum albumin (Sigma) dissolved in Tris-buffered saline with 0.1% Tween.

Primary mouse monoclonal antibodies were: cTnC (1∶5000 dilution; Santa Cruz Biotechnology, #sc-48347); cTnT (1∶2500; Abcam, #ab8295); smooth muscle MLCK (1∶5000, Sigma Aldrich, #m7905). Primary rabbit polyclonal antibodies were from Abcam: p-cTnI (Ser43) (1∶500, #ab59420); p-cMLC2 (Ser20) (1∶1000, #ab2480); cMLC2 (1∶10000, #ab92721); or Cell Signalling: p-cTnI (Ser23/24) (1∶1500, #4004); cTnI (1∶1500, #4002); p-MYPT1 (Thr696) (1∶750, #ab545); MYPT1(1∶1000, #cs-2634); PKCα (1∶1000, #2056); PKCε (1∶1000, #26831); RhoA(67B9) (1∶1000, #21175). GAPDH (1∶3000; Abcam, #ab9485) was used to standardize for loading. Horseradish peroxidase-conjugated goat anti-mouse (1∶5000) or anti-rabbit (1∶10000) secondary antibodies (Abcam, MA) were used at room temperature for 1 hour. Immunologic detection was accomplished using Amersham ECL Western blotting detection reagents (GE Healthcare). Protein levels were quantified by densitometry using NIH ImageJ analysis software.

### RhoA Activity

A G-LISA kit was used (BK124; Cytoskeleton Inc., CO). After lysis in a buffer containing 50 mM NaF (Sigma Aldrich), 20 mM sodium pyrophosphate (Sigma Aldrich) and 1 mM p-nitrophenyl phosphate (Merck Chemicals) to block phosphatase activity, homogenization, and protein concentration quantification, aliquots of lysate (0.5 mg protein/ml) were added to wells linked to a Rho-GTP-binding protein. Active (GTP-bound) RhoA attached to the wells. Inactive (GDP-bound) RhoA was eluted. Active RhoA was detected with an anti-RhoA antibody, and absorbance read at 490 nm with a PHERAStar FS microplate reader (BMG, Germany).

### Statistical Analyses

All experiments and analyses were blinded. In skinned fibers, relative force was plotted against pCa (−log_10_ [Ca^2+^]) and fitted with Hill plots to determine pCa_50_ (the pCa at half-maximal force) and Hill coefficient values [6]. In isolated hearts, the maximum rate of change of left ventricular pressure (dP/dt_max_) was scaled as a % of baseline, and data from individual experiments fitted to a non-linear regression equation (Statistica programme, StatSoft, Tulsa, USA) to determine EC_25_, EC_50_ and EC_75_, the agonist concentrations generating 25%, 50% and 75% of the maximum dP/dt_max_ response, respectively, and analyzed using one-way ANOVA. Data points from different dose-response curves were compared using two-way ANOVA for repeated measures, with Newman-Keuls post-hoc test when indicated. For protein expression quantification, unpaired *t* tests were used for comparisons between two groups, and two-way ANOVA with multiple comparisons was used among groups (GraphPad Prism version 6.00 for Windows, GraphPad Software, California, USA). Results are mean ±1 SEM.

## Results

### CM Morphology

**Figure 1:**
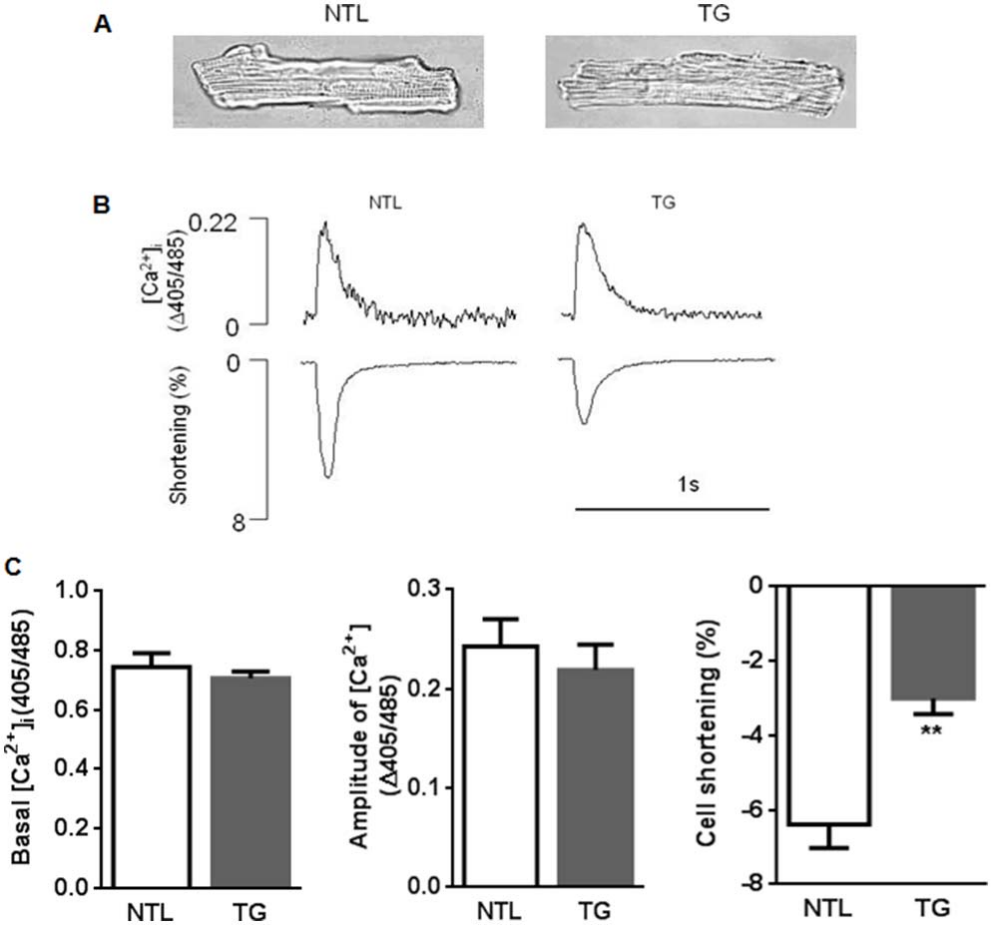
Baseline characteristics of α_1A_-TG cardiomyocytes. A, representative images of single CMs. **B,** representative recordings of Ca^2+^ transients and percent cell shortening. **C,** composite data for basal [Ca^2+^]_i_, amplitude of the systolic [Ca^2+^]_i_ rise and CM cell shortening in NTL (n = 7) and α_1A_-TG (n = 7) hearts. Data are shown as the mean ± SEM. **P<0.01 vs. NTL.

Cell morphology did not differ between α_1A_-TG and NTL CMs (Fig. 1A and Table 1), confirming the absence of hypertrophy with α_1A_-AR overexpression [1].

**Table 1:** Cardiac myocyte characteristics.

Parameters	NTL (n = 11)	α_1A_-TG (n = 9)
Myocyte Length (µm)	97±2.7	99±2
Myocyte Width (µm)	21±0.6	21±0.3
Myocyte Perimeter (µm)	236±5.5	240±5
Myocyte Area (µm^2^)	2065±77	2091±85
Length/Width Ratio	4.79±0.16	4.89±0.1
Sarcomere Length (µm)	1.58±0.002	1.61±0.006
Sarcomere Number	59±1.5	56±1.7

Data shown are mean ± SEM. n is the number of hearts, with 5 cells measured per heart, on average.

### α_1A_-TG CMs Exhibit Hypocontractility in the Absence of Agonist Stimulation

**Figure 2:**
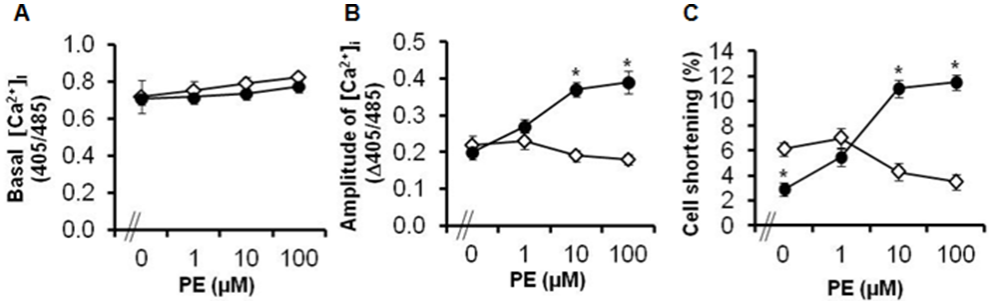
Contractility in α_1A_-TG cardiomyocytes. Indices of excitation-contraction coupling before and after α_1A_-AR agonist stimulation with phenylephrine (PE) in NTL (◊, n = 7) and α_1A_-TG (•, n = 7) CMs. **A,** basal [Ca^2+^]_I_; **B,** amplitude of the systolic [Ca^2+^]_i_ rise (Peak-Basal); **C,** percent cell shortening. Data are shown as the mean ± SEM. *P<0.05 vs. NTL.

In the absence of α_1A_-AR agonist, α_1A_-TG CMs exhibited *reduced* shortening (Fig. 1B, *P*<0.01). This unexpected finding was not associated with a reduction in either resting [Ca^2+^]_i_ or the systolic amplitude of the [Ca^2+^]_i_ transient, which reflects Ca^2+^ released from the sarcoplasmic reticulum (Fig. 1B, 1C and Fig. 2). Kinetic studies showed no changes in Ca^2+^ release or reuptake rates in α_1A_-TG CMs (data not shown).

### α_1A_-TG CMs are Hypersensitive to α_1A_-AR Agonist Stimulation

Despite the reduced shortening of α_1A_-TG CMs observed in the absence of agonist stimulation, concentration-response curves to incremental doses of the α_1_-AR agonist, PE, demonstrated greater shortening of α_1A_-TG than NTL CMs (Fig. 2C). The hypersensitivity of the shortening response to PE in α_1A_-TG CMs paralleled increased Ca^2+^ release with increasing agonist stimulation in these CMs, as reflected by the higher amplitude of the systolic rise in [Ca^2+^]_i_ (Fig. 2B). In contrast, NTL CMs did not demonstrate any significant change in either shortening or the amplitude of the [Ca^2+^]_i_ transient in response to PE. Resting [Ca^2+^]_i_ did not increase significantly with PE in α_1A_-TG or NTL CMs (Fig. 2A).

### α_1A_-TG Isolated Perfused Hearts Exhibit Hypocontractility in the Absence, and Hypercontractility in the Presence of Agonist Stimulation

**Figure 3:**
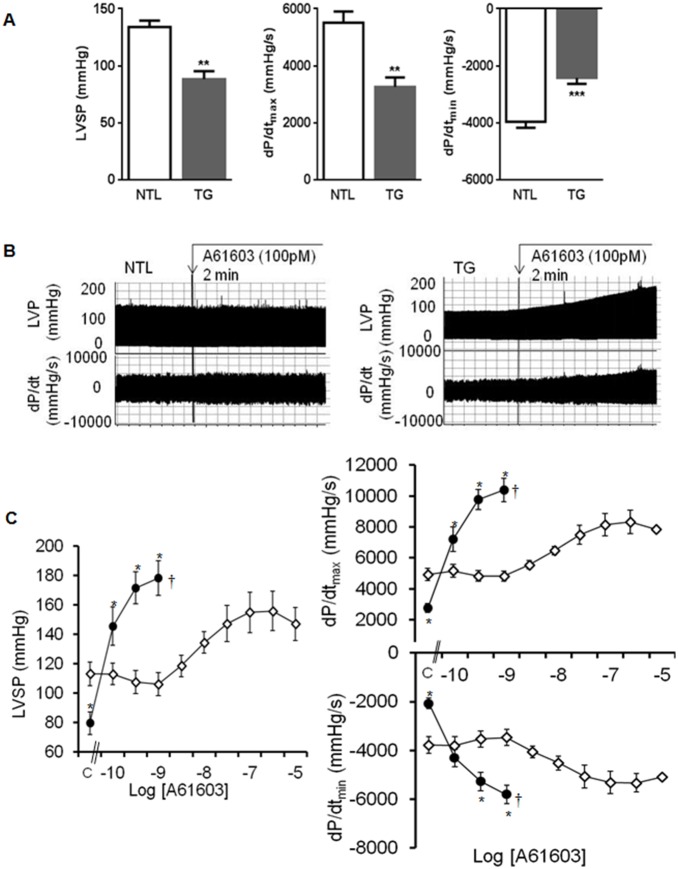
Contractility in α_1A_-TG isolated working hearts. **A,** baseline left ventricular systolic pressure (LVSP), dP/dt_max_ and dP/dt_min_ of isolated perfused contracting NTL (n = 17) and α_1A_-TG (n = 24) hearts. **B,** representative recordings of left ventricular pressure (LVP) and dP/dt at baseline and during A61603 infusion (100 pM). **C,** composite data obtained from NTL (◊, n = 6) and α_1A_-TG (•, n = 7) hearts at baseline (C) and dose-response to A61603. Data are shown as the mean ± SEM. *P<0.05, **P<0.01; ***P<0.001 vs. NTL.

The isolated CM experiments suggested that the hypercontractility of α_1A_-TG hearts *in vivo* might reflect hypersensitivity to endogenous catecholamines. To evaluate responses in the intact organ, we tested isolated perfused contracting heart preparations and found responses (Fig. 3) that closely mirrored those observed in isolated CMs. In the absence of agonist, isolated α_1A_-TG hearts exhibited significantly reduced contractility (Fig. 3A), evidenced by lower peak pressure generation and lower dP/dt_max_, as well as impaired relaxation (dP/dt_min_). Heart rate (α_1A_-TG 381±17 vs. NTL 371±14 bpm) and coronary flow (2.5±0.2 vs. 2.2±0.1 ml/min) were not different.

With the selective α_1A_-AR agonist, A61603, the α_1A_-TG hearts demonstrated marked hypercontractility, evidenced by higher peak pressure and higher dP/dt_max_ for any given concentration of A61603, with parallel increments in dP/dt_min_ (Fig. 3B, 3C). EC_50_ was significantly lower in α_1A_-TG hearts (0.082±0.003 nM vs. NTL 10.1±2.8 nM, *P*<0.05). A61603 concentrations above 3 nM caused rapid decompensation in α_1A_-TG hearts, corresponding to the sudden death phenotype documented previously *in vivo* [5].

### Baseline Hypocontractility in α_1A_-TG Hearts is not due to Heterologous Desensitization

**Figure 4:**
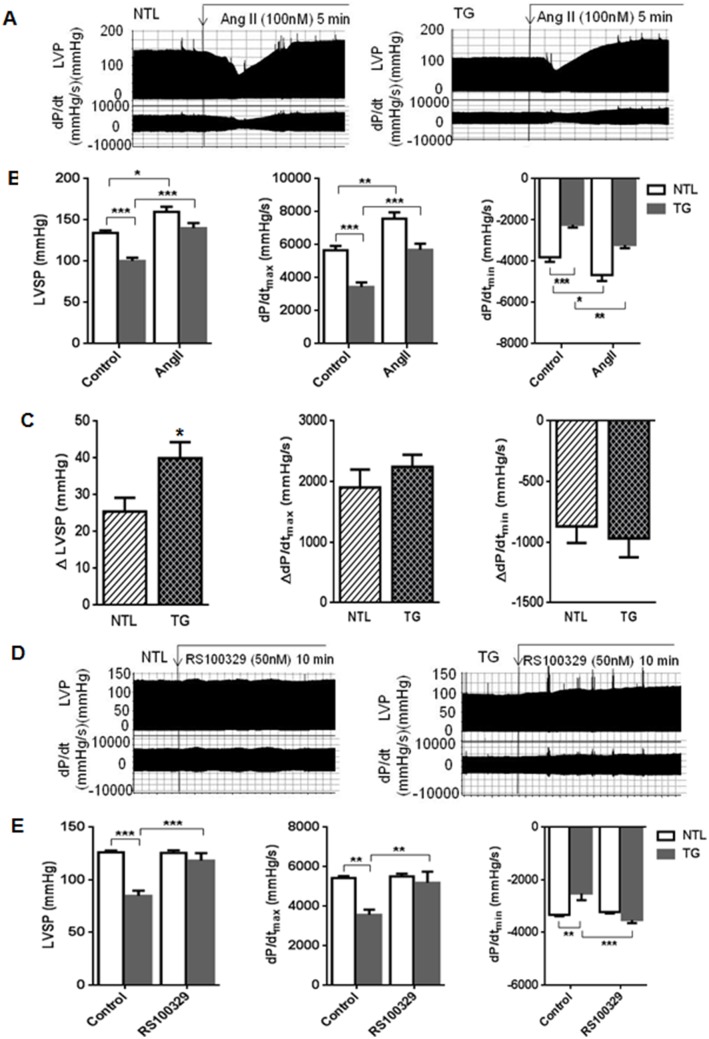
Hypocontractility in α_1A_-TG hearts is not due to heterologous desensitization but is mediated by the α_1A_-AR. **A,** representative recordings of left ventricular pressure (LVP) and dP/dt at baseline and during AngII infusion (100 nM) in isolated perfused contracting hearts. **B,** composite data at baseline (Control) and after AngII infusion (100 nM) for 10 min in NTL (□, n = 7) and α_1A_-TG (▪, n = 9) hearts; **C,** change (Δ) from baseline for B; **D,** representative recordings of LVP and dP/dt at baseline and during α_1A_-AR selective antagonist, RS100329, infusion (50 nM); **E,** composite data at baseline (Control) and after RS100329 infusion (50 nM) for 10 min in NTL (□, n = 5) and α_1A_-TG (▪, n = 4) hearts. Data are shown as the mean ± SEM. *P<0.05, **P<0.01, ***P<0.001.

One possible explanation for baseline α_1A_-TG hypocontractility is that sustained overstimulation of the contractile apparatus, due to activation of the greatly increased number of α_1A_-ARs by endogenous catecholamines, results in heterologous downregulation of contractility at a sub-receptor level (that is, desensitization to multiple agonists resulting from excessive exposure to a single stimulus). If so, stimulation of the *same* sub-receptor pathway via an *alternate* Gα_q_-linked receptor would be expected to elicit a reduced contractile response in α_1A_-TG hearts. To test this hypothesis, isolated perfused hearts were treated with AngII to activate the Gα_q/11_-coupled AT_1_ receptor. AngII produced a transient negative, followed by a large sustained positive inotropic response (Fig. 4A−4C). The positive inotropic effect of AngII, the increment in peak pressure or dP/dt_max_ from baseline, was not reduced in α_1A_-TG hearts (Fig. 4C).

### Baseline Hypocontractility in α_1A_-TG Hearts is Mediated by the α_1A_-AR

To test the alternative hypothesis that the hypocontractility observed in α_1A_-TG hearts in the absence of agonist stimulation is mediated by the α_1A_-AR, isolated perfused contracting hearts from α_1A_-TG and NTL mice were perfused with the selective α_1A_-AR antagonist, RS100329. RS100329 completely reversed the hypocontractility and impaired relaxation observed in α_1A_-TG hearts within 10 min of treatment onset (Fig. 4D, 4E). To ensure that this dramatic effect of RS100329 was indeed due to its specific antagonism of the α_1A_-ARs, we repeated these experiments with a *different* selective α_1A_-AR antagonist, KMD3213 dihydrobromide, and again demonstrated complete reversal of the hypocontractility and impaired relaxation observed in α_1A_-TG hearts within 10 min of treatment onset (Fig. S1).

### α_1A_-AR Overexpression does not Alter Ca^2+^ Sensitivity in Skinned Cardiac Fibers

Next we evaluated myofilament Ca^2+^ sensitivity by measuring steady-state isometric force development in skinned cardiac fibers from α_1A_-TG and NTL ventricular strips. Stepped changes in pCa produced increments in steady-state force, but the resulting composite Hill curves quantifying the force-Ca^2+^ relationship were not significantly different for α_1A_-TG and NTL ventricular strips (n = 13): pCa_50_ 5.86±0.12 α_1A_-TG vs. 5.88±0.11 NTL; Hill coefficient 3.03±0.12 nCa α_1A_-TG vs. 2.82±0.19 nCa NTL. The pCa_50_ is a measure of the sensitivity of the contractile apparatus to Ca^2+^. The Hill coefficient gives an indication of the affinity of the functional unit of the contractile apparatus for Ca^2+^.

### α_1A_-AR Overexpression Reduces Phosphorylation of cMLFC2 by Inhibiting RhoA Activity

**Figure 5:**
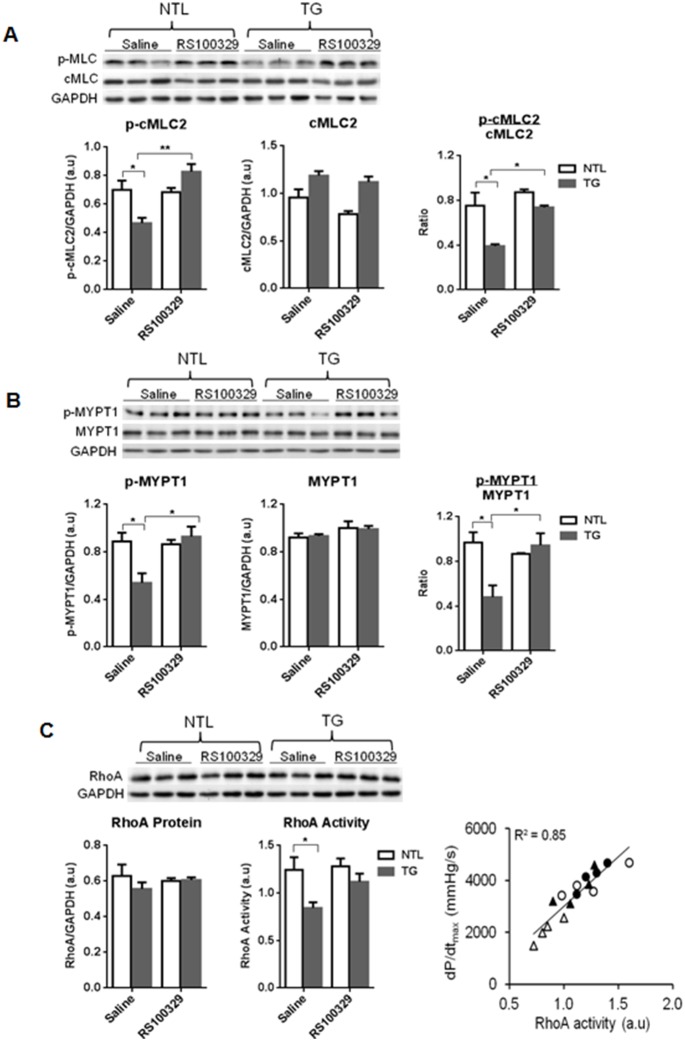
Mechanism of α_1A_-TG hypocontractility. Western blot analyses of myofilament proteins and RhoA activity in NTL (□) and α_1A_-TG (▪) hearts after infusion of saline or RS100329 (50 nM) for 8 min. In each panel, representative Western blots and pooled data (n = 3/group) are shown: **A,** p-cMLC2(Ser20), total cMLC2, and their ratio; **B,** p-MYPT1(Thr696), total MYPT1, and their ratio; **C,** RhoA protein expression, RhoA activity and the relationship between dP/dt_max_ and RhoA activity, where data are shown from NTL isolated hearts treated with saline (○) or RS100329 (•) and α_1A_-TG hearts treated with saline (Δ) or RS100329 (▴). Western blot data are normalized to GAPDH expression. Data are shown as the mean ± SEM. *P<0.05, **P<0.01.

Although myofilament Ca^2+^ sensitivity was not different in α_1A_-TG skinned cardiac fibers, a modulating effect of myofilament protein phosphorylation on Ca^2+^ sensitivity *in vivo,* where the cell membrane is intact, could not be excluded. To address this issue, we assessed the phosphorylation status of the key contractile proteins, cTnI and cMLC2, in isolated hearts from α_1A_-TG and NTL mice perfused with vehicle (saline) or RS100329. There were no differences in total cTnI or p-cTnI, or in cTnC or cTnT, between α_1A_-TG and NTL hearts (data not shown), but α_1A_-TG mice exhibited significant hypophosphorylation of cMLC2 (Fig. 5A). Importantly, the decreases in p-cMLC2 and in the ratio of p-cMLC2/cMLC2 were rapidly reversed by the selective α_1A_-AR antagonist, RS100329, within 8 minutes.

PKC, MLCK and MLCP regulate MLC phosphorylation in smooth muscle. PKCα was significantly higher in α_1A_-TG (PKCα/GAPDH 1.0±0.08) than NTL hearts (0.71±0.08, *P*<0.05), but was not altered by RS100329 treatment. PKCε was unaffected by genotype or treatment (data not shown). MLCK phosphorylates MLC in response to changes in [Ca^2+^]_i_, but was unaffected by genotype or RS100329 treatment (data not shown), consistent with the fact that baseline hypocontractility in α_1A_-TG hearts was not associated with changes in [Ca^2+^]_i_.

Conversely, dephosphorylation of MLC is mainly catalyzed by MYPT1, a myosin binding regulatory subunit of MLCP. The level of MYPT1 phosphorylated at Thr696, but not Thr853, was significantly reduced in α_1A_-TG mice (Fig. 5B), and this was rapidly reversed by RS100329.

Given that active (that is, GTP-bound) RhoA binds to the C-terminal region of MYPT1, and that activated ROCK inhibits MLCP by phosphorylating MYPT1 at Thr696, we next examined RhoA/ROCK signaling. RhoA activity was significantly reduced in α_1A_-TG hearts (Fig. 5B), a reduction rapidly reversed by RS100329, but protein expression of RhoA (Fig. 5A), or of ROCK1 or ROCK2 (data not shown), was unchanged. Cardiac contractility (dP/dt_max_) was directly correlated with RhoA activity (R^2^ = 0.85, Fig. 5C).

### RhoA/ROCK Signaling Maintains Basal Cardiac Contractility in the Normal Heart

**Figure 6:**
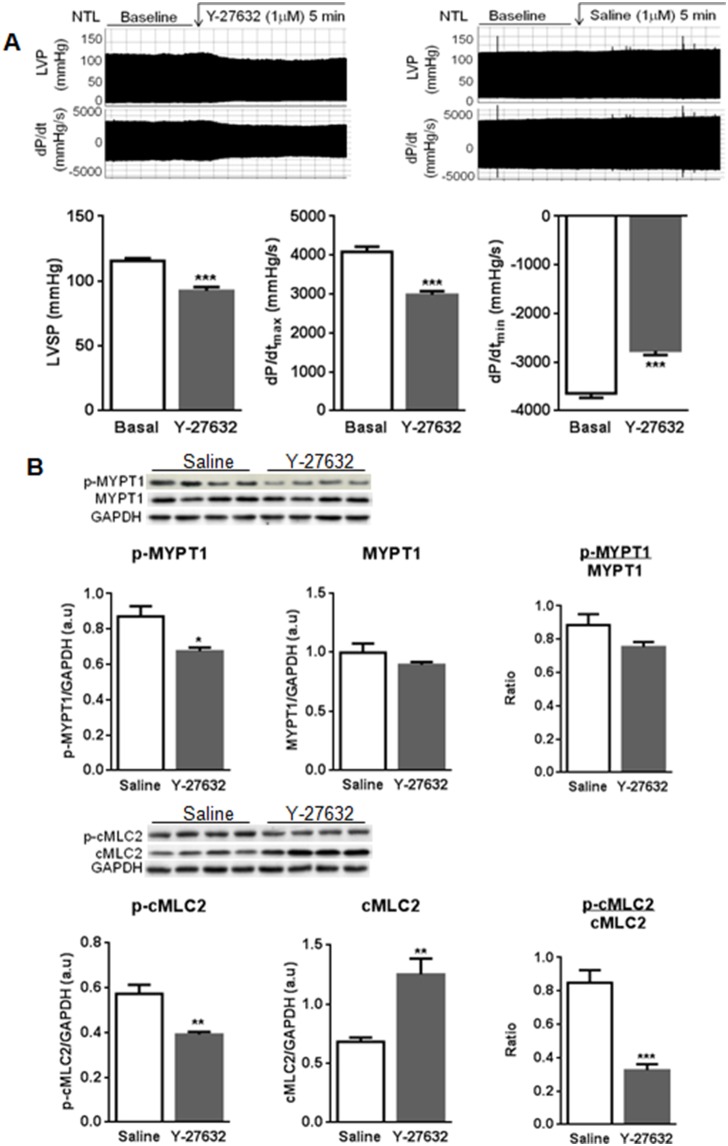
RhoA mediates basal cardiac contractility in normal mice. **A,** representative recordings of left ventricular pressure (LVP) and dP/dt at baseline and during saline or Y-27632 infusion (1 µM) for 5 min in NTL hearts (top panel); composite data (n = 7, bottom panel); **B,** representative Western blots (top panel) and pooled data (n = 4/group) normalized for GAPDH loading, showing p-MYPT1(Thr696), total MYPT1, and their ratio (middle panel) and p-cMLC2(Ser20), total cMLC2, and their ratio (bottom panel). Data are shown as the mean ± SEM. *P<0.05, **P<0.01, ***P<0.001 vs. control.

To further evaluate the involvement of RhoA/ROCK signaling in basal contractility, hearts were treated with Y-27632, a selective ROCK inhibitor. Selective ROCK inhibition caused significant falls in peak pressure, dP/dt_max_ and dP/dt_min_ in NTL hearts (Fig. 6A) within 5 minutes, accompanied by significant falls in the level of MYPT1 phosphorylated at Thr696 and p-cMLC2 (Fig. 6B), but caused no further reduction in basal contractility in α_1A_-TG hearts, and had no effect on the increased contractility with A61603 in either NTL or α_1A_-TG hearts (data not shown).

## Discussion

### Mechanism of Increased Contractility without Hypertrophy with α_1A_-AR Overexpression

We demonstrated that the mechanism of increased contractility with α_1A_-AR overexpression was increased intracellular Ca^2+^ release in response to agonist stimulation. This was not unexpected because other Gα_q/11_-coupled receptors, including the AT_1_ and endothelin receptors, increase Ca^2+^ release by activating phospholipase Cβ, and this would account for the increased PKCα expression we observed. In addition, α_1A_-AR coupled Ca^2+^ entry depends on a novel mechanism involving redirection and activation of the transient receptor potential canonical 6 (TRPC6) channel from the cytoplasm to the plasma membrane via interaction with Snapin, but α_1A_-AR activation of Gα_q/11_ also produces diacylglycerol that independently activates TRPC6 in the plasma membrane [7]. Activation of the greatly increased number of α_1A_-ARs by endogenous catecholamines could thus account for the hypercontractility seen *in vivo* [1], but the elevated [Ca^2+^]_i_ might be expected to stimulate cardiac hypertrophy also. Marked hypertrophy is observed, for example, in mice with cardiac overexpression of Gα_q_ [8] or other Gα_q/11_-coupled receptors, such as the AT_1_ receptor [9], yet hypertrophy was not evident in our α_1A_-TG CMs or in mouse or rat hearts *in vivo* [1], [10]. This may be because α_1A_-AR activation *in vivo* is not sustained but intermittent, fluctuating with endogenous catecholamine levels. This is consistent with the propensity of α_1A_-TG mice to stress-related sudden cardiac death suggestive of Ca^2+^ overload [5]. Sustained α_1A_-AR activation would be expected to cause heterologous desensitization of the contractile response [11], but we found no evidence of this. Despite the large increase in the systolic *amplitude* of the [Ca^2+^]_i_ transient with agonist stimulation of α_1A_-TG CMs, we observed no change in *resting* [Ca^2+^]_i_ with repeated but non-sustained α_1A_-AR activation (Fig. 2C), which could account for the lack of hypertrophy.

### Mechanism of Reduced Contractility with α_1A_-AR Overexpression in the Absence of Agonist

The unexpected finding in our study was the *reduced* contractility observed with α_1A_-overexpression in the absence of agonist. Overexpression of other G protein-coupled receptors, such as the β-AR, results in marked agonist-independent receptor signaling due to spontaneous receptor isomerization [12]. The hypocontractility with α_1A_-AR overexpression was not due to any alteration in [Ca^2+^]_i_. Nor was the hypocontractility due to heterologous desensitization, as noted above. We also demonstrated that the sensitivity of the contractile machinery to Ca^2+^ was unaltered in α_1A_-TG skinned cardiac fibers, but this preparation is minimally phosphorylated [13].

We explored whether myofilament Ca^2+^ sensitivity was impaired due to altered phosphorylation. In cardiac muscle, Ca^2+^ sensitivity is thought to be regulated mainly by the troponin complex, but we found no alterations in the cardiac troponins or their phosphorylation status. In smooth muscle, contraction is primarily dependent on phosphorylation of regulatory MLC, which is controlled by the opposing activities of Ca^2+^/calmodulin-dependent MLCK and Ca^2+^-independent MLCP. Moreover, activation of the small GTPase, RhoA, and its downstream target, ROCK, results in Ca^2+^ sensitization as a result of MYPT1 phosphorylation and, thus, inhibition of MLCP, increasing MLC phosphorylation in smooth muscle [14]. Phosphorylated MLC binds to myosin at the head-rod junction, which facilitates actin-myosin interactions that enhance contractility.

Our major finding was that the reduced cardiac contractility with α_1A_-TG overexpression was due to cMLC2 hypophosphorylation. We explored whether this was driven by alterations in MLCK or the RhoA/ROCK signaling pathway. Because there was no change in [Ca^2+^]_i_, the absence of any change in expression of the Ca^2+^/calmodulin-dependent MLCK was expected. The significant hypophosphorylation of cMLC2 was due to reduced RhoA activity and reduced phosphorylation of MYPT1. RhoA activity was strongly correlated with cardiac contractility. Importantly, the hypocontractility and all of the changes in the RhoA/ROCK signaling pathway were rapidly reversed by selective α_1A_-AR blockade. In contrast, the increased PKCα expression we observed in α_1A_-TG hearts, which could conceivably have contributed to the hypocontractility [15], was unchanged with selective α_1A_-AR blockade.

### Pleiotropic Signaling by the α_1A_-AR

The rapid reversal of the agonist-independent hypocontractility in α_1A_-TG hearts after selective α_1A_-AR blockade with two different selective antagonists indicates that the hypocontractility results from spontaneous receptor activity. But the activated states in the absence and presence of agonist are *different*: *hypocontractility* in the absence but *hypercontractility* in the presence of agonist. These effects cannot be explained by promiscuous coupling to extraneous pathways as a result of α_1A_-AR overexpression because the α_1A_-AR used to develop the α_1A_-TG model was the wild type, not a mutant [1].

**Figure 7:**
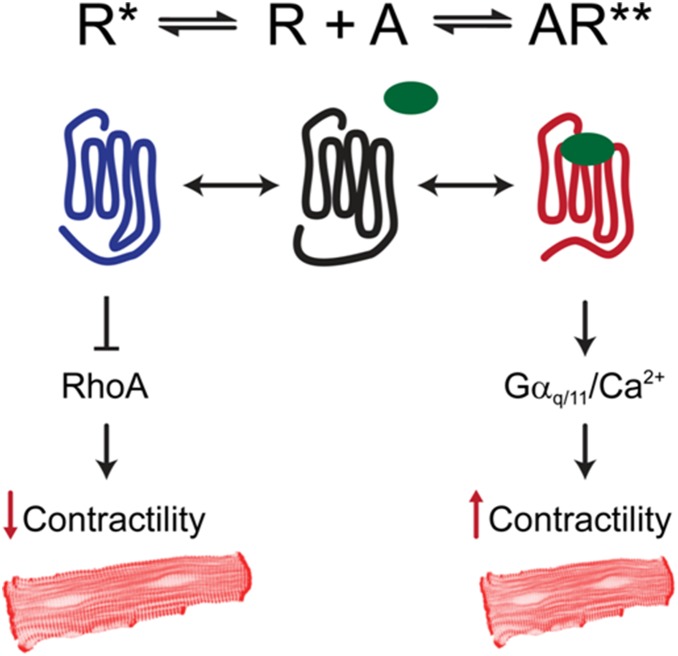
Proposed model of pleiotropic α_1A_-AR signaling effects on contractility. Schematic outlining how distinct conformations of the α_1A_-AR could lead to the opposing physiological effects of hypo- and hypercontractility that were observed in α_1A_-AR TG mice. R* is the conformation of the receptor that, in the absence of ligand (A), constitutively suppresses RhoA activity, leading to hypocontractility. Conversely, agonist-bound α_1A_-ARs (R**) adopt a distinct conformation that signals via Gα_q/11_ and Ca^2+^ to enhance CM contractility.

We propose a model of pleiotropic receptor signaling (Fig. 7) in which contractility is suppressed by engagement of the agonist-independent activated conformation of the receptor (R*) with the RhoA/ROCK pathway, leading to its inhibition. In contrast, agonist activation of the receptor induces a distinct active conformation (R**) that does not involve engagement of the RhoA/ROCK pathway but enhances contractility by both α_1A_-AR coupled Ca^2+^ entry [7] and Gα_q/11_-dependent Ca^2+^ release. We have shown previously that a single receptor subtype can adopt differing activated conformations to engage distinct downstream signaling pathways [16], [17]. How R* suppresses RhoA/ROCK signaling is presently being investigated, but the rapid reversal after selective α_1A_-AR blockade points to altered protein activation rather than expression. Potential mechanisms include activation of a RhoA guanine nucleotide dissociation inhibitor (RhoGDI) [18], either directly or by initial interaction of R* with a β-arrestin, perhaps by activating a kinase that phosphorylates RhoGDI, or inhibits a GDI displacement factor that mediates RhoA.RhoGDI dissociation.

### Physiological Role of RhoA/ROCK Signaling and Clinical Implications

The link between cardiac contractility and RhoA/ROCK signaling in animals with 170-fold overexpression of the α_1A_-AR raises the question of physiological relevance. Although contractility is reduced in mice with a non-phosphorylatable form of cMLC2, and reduced phosphorylation of cMLC2 has been found in failing human and mouse hearts [19], [20], a physiological role for cMLC2 in regulating cardiac contractility has not been clearly established. Similarly, chronic inhibition of the RhoA/ROCK pathway may prevent adverse remodeling in experimental heart failure models [21], [22], but its physiological role in regulating contractility remains unclear. ROCK inhibition has been reported to decrease endothelin-1 induced increases in contractility in rabbit ventricular CMs [23], but others have reported enhanced cardiac contractility after ROCK inhibition in infarct and diabetic experimental models [24], [25].

To address this issue more directly, we examined ROCK inhibition in NTL hearts with normal α_1A_-AR expression, demonstrating a significant reduction in baseline contractility in association with reduced phosphorylation of MYPT1 and cMLC2. These findings indicate that the RhoA/ROCK pathway plays an important physiological role in maintaining normal baseline contractility. This normal role may be amplified in heart failure, when the β-ARs are downregulated and uncoupled from G proteins, and with the increasing therapeutic use of β-AR blockers. Moreover, increased contractility with RhoA/ROCK pathway activation does not depend on increased Ca^2+^ release, suggesting it as a promising target for development of novel inotropic agents that might not increase mortality with long term use.

### Limitations

Our novel finding of depressed cardiac contractility due to agonist-independent activity of the α_1A_-AR is based on a model with 170-fold overexpression of the receptor. Nevertheless, this model has allowed us to identify pleiotropic signaling by the receptor that may have broader significance for receptor physiology. As noted above, promiscuous coupling due to receptor overexpression can be excluded because the model is based on the wild type α_1A_-AR. This model also allowed us to identify the importance of RhoA/ROCK signaling and its control of MLC2 phosphorylation in modulating cardiac contractility, and we have demonstrated that this mechanism supports baseline contractility even in the setting of normal α_1A_-AR expression. The mechanism by which the α_1A_-AR inhibits RhoA activity in the absence of ligand remains to be determined in future experiments.

## Supporting Information

Figure S1 **Hypocontractility and impaired relaxation in α_1A_**-**TG hearts are reversed with the selective α_1A_**-**AR antagtonist, KMD3213. A,** representative recordings of left ventricular pressure (LVP) and dP/dt at baseline and during KMD3213 infusion (100 nM) in isolated perfused contracting hearts. **B,** composite data at baseline (Control) and after KMD3213 infusion (100 nM) for 10 min in NTL (n = 4) and α_1A_-TG (n = 4) hearts. Data are shown as the mean ± SEM. *P<0.05, **P<0.01.
(TIF)
