# Supplementary figures and images for: RhoA/ROCK Signaling and Pleiotropic α1A-Adrenergic Receptor Regulation of Cardiac Contractility
Source: PLoS One. 2014 Jun 11;9(6):e99024. doi: 10.1371/journal.pone.0099024 (PMC4053326; doi:10.1371/journal.pone.0099024)

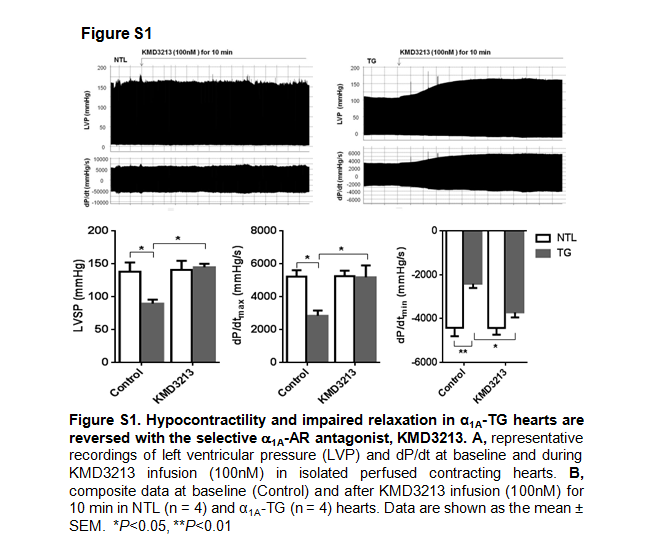

Supplement: Figure S1 — Hypocontractility and impaired relaxation in α1A-TG hearts are reversed with the selective α1A-AR antagtonist, KMD3213. A, representative recordings of left ventricular pressure (LVP) and dP/dt at baseline and during KMD3213 infusion (100 nM) in isolated perfused contracting hearts. B, composite data at baseline (Control) and after KMD3213 infusion (100 nM) for 10 min in NTL (n = 4) and α1A-TG (n = 4) hearts. Data are shown as the mean ± SEM. *P<0.05, **P<0.01. (TIF) [file pone.0099024.s001.tif]
